# Establishment of a risk prediction model for bowel preparation failure prior to colonoscopy

**DOI:** 10.1186/s12885-024-12081-4

**Published:** 2024-03-14

**Authors:** Na Zhang, Miaomiao Xu, Xing Chen

**Affiliations:** 1 The First Hospital of Shanxi Medical University, 030000 Taiyuan, Shanxi Province China; 2 Tongji Medical College, The Central Hospital of Wuhan, Huazhong University of Science and Technology, 430000 Wuhan, Hubei Province China

**Keywords:** Colonoscopy, Bowel preparation, Influencing factors, Prediction model

## Abstract

**Background:**

This study aimed to determine the factors that contribute to the failure of bowel preparation in patients undergoing colonoscopy and to develop a risk prediction model.

**Methods:**

A total of 1115 outpatients were included. Patients were randomly divided into two groups: the modeling group (669 patients) and the validation group (446 patients). In the modeling group, patients were further divided into two groups based on their success and failure in bowel preparation using the Boston Bowel Preparation Scale. A logistic regression analysis model was used to determine the risk factors of bowel preparation failure, which was subsequently visualized using an alignment diagram.

**Results:**

After controlling for relevant confounders, multifactorial logistic regression results showed that age ≥ 60 years (*OR* = 2.246), male (*OR* = 2.449), body mass index ≥ 24 (*OR* = 2.311), smoking (*OR* = 2.467), chronic constipation (*OR* = 5.199), diabetes mellitus (*OR* = 5.396) and history of colorectal surgery (*OR* = 5.170) were influencing factors of bowel preparation failure. The area under the ROC curve was 0.732 in the modeling group and 0.713 in the validation group. According to the calibration plot, the predictive effect of the model and the actual results were in good agreement.

**Conclusions:**

Age ≥ 60 years, male, body mass index ≥ 24, smoking, chronic constipation, diabetes mellitus, and history of colorectal surgery are independent risk factors for bowel preparation failure. The established prediction model has a good predictive efficacy and can be used as a simple and effective tool for screening patients at high risk for bowel preparation failure.

## Background

Colorectal cancer is a common malignant tumor that ranks third in incidence and second in mortality in the latest global statistics on tumors [1]. There is evidence that early detection and treatment of precancerous lesions, such as colorectal adenomas, can significantly reduce colorectal cancer incidence and mortality [2, 3]. Colonoscopy is the most effective method of screening for colorectal cancer. Successful bowel preparation is an essential part of colonoscopy, and the quality of bowel preparation is clearly required by international guidelines that bowel preparation failure rate should be under 10% for endoscopic diagnosis and treatment [4]. However, the reported failure rate of bowel preparation in patients undergoing colonoscopy is as high as 18-35% [5, 6]. The failure of bowel preparation renders colonoscopy difficult, prolongs the examination time, increases misdiagnosis and complications, and shortens the interval between procedures, subsequently increasing patient pain and medical costs [7, 8]. Therefore, it is crucial to identify and prevent bowel preparation failure as early as possible.

Demographic characteristics and living habits vary greatly among countries. Based on a meta-analysis of subgroups in Asian and Western countries, it was determined that factors influencing the quality of bowel preparation differed across ethnic and regional groups [6]. Although several studies from western countries have developed predictive models for the risk of bowel preparation failure [9–11], few studies in China have focused on this topic. Therefore, it is imperative to develop a predictive model based on our national population with Chinese standard. In this study, we analyzed the risk factors for bowel preparation failure and developed an intuitive and convenient alignment diagram for predicting the risk of bowel preparation failure.

## Methods

### Study population

Outpatients over 18 years who underwent colonoscopy in the First Hospital of Shanxi Medical University from March 2022 to July 2022 were selected as the study subjects. Exclusion criteria included those who were had communication problems; poor compliance with the bowel preparation protocol (poor compliance is indicated if the dose of medication was < 75% of the prescribed dose, time of taking medication was wrong, or high-fiber diet was consumed 24 h before the examination), emergent colonoscopies and interruption of the examination owing to intestinal obstruction or other reasons not related to bowel preparation. Informed written consent was obtained from all participating patients. The Ethical Committee and Institutional Review Board of the First Hospital of Shanxi Medical University reviewed and approved this study protocol.

By combining the results of expert consultation with the findings of our previous meta-analysis [12] of individual factors influencing the colonoscopy bowel preparation quality, we developed our own questionnaire. The contents included age, sex, education level, body mass index (BMI), dietary preferences, exercise habits, smoking history, drinking history, defecation habits, comorbidities, previous medication history, previous surgery history, indication, history of failed bowel preparation, dosage of polyethylene glycol electrolyte solution, and type of diet during bowel preparation. Old age was defined as over 60 years and overweight was defined as BMI over 24 kg/m^2^ according to official Chinese standard [13–14]. The dietary preferences of patients were divided into three categories: meat-based, vegetarian-based, and meat-vegetarian combination. Patients were categorized according to the quantity of meat and vegetarian foods they consumed every day in their diet. Generally, a diet containing vegetarian (or meat) food is vegetarian (or meat)-based if the proportion of vegetarian (or meat) food exceeds 50%, and a diet that contains both in proportion of 50% is considered meat-vegetarian combination. Based on the frequency of patients’ exercise, we classified exercise habits into three categories: never, occasionally, and regularly. The never exercise category was defined as no exercise, occasional exercise implied at least one exercise per month, and regular exercise implied at least one exercise per week. History of alcohol consumption was defined as average intake of alcohol > 50 g/d for more than 1 year or abstinence from alcohol less than 1 year. History of smoking was defined as average smoking > 1 cigarette/d, and continuous smoking > 1 year, or those who had quit for less than 1 year. Researchers conducted a one-on-one interview with the patients on the day of the colonoscopy and recorded the information according to the patient’s responses.

### Bowel preparation method

Two researchers instructed all patients in this study regarding the use of a split oral 3 L polyethylene glycol electrolyte powder bowel preparation protocol. A day prior to the colonoscopy, a low-fiber diet was consumed, and fasting was observed on the day of the procedure. A sachet of polyethylene glycol electrolyte powder dissolved in 1,000 ml of warm water was administered orally at 19:00 a day before the examination. Two sachets of polyethylene glycol electrolyte dispersion dissolved in 2,000 ml of warm water were administered orally 4–6 h before the examination, and 30 ml of dimethicone emulsion was administered at the same time as the last laxative. Patients were instructed to drink each bag of laxatives within 1 h, either 250 ml every 15–20 min in four divided doses at a slow rate to avoid nausea, vomiting or other discomforts. In addition, patients were instructed to rub the abdomen gently and walk around appropriately during the dosing period.

### Bowel preparation quality score

The endoscopist used the Boston Bowel Preparation Scale (BBPS) [15] to score the cleanliness of the three colonic segments of the patient’s right colon (including ileocecal and ascending colon), transverse colon (including hepatic flexure and splenic flexure), and left colon (including descending colon, sigmoid colon, and rectum). The scoring criteria were as follows: 0, a solid stool could not be removed from the intestinal cavity and the entire intestinal mucosa was not visible; 1, the intestinal cavity was still filled with stool or dark liquid, and part of the intestinal mucosa was visible; 2, a small amount of stool or dark liquid remained in the intestinal cavity and the intestinal mucosa was visible; 3, the intestinal cavity did not contain any stool or dark liquid and the entire intestinal mucosa was visible. The total score was the sum of the three colonic segments scores, and a total score of ≥ 6 and a score of ≥ 2 for any 1 colonic segment was considered successful bowel preparation. A total score of < 6 or a score of < 2 for any one colonic segment was considered failed colonic preparation.

### Statistical analysis

The SPSS 26.0, R4.1.1 software was used for statistical analysis. Quantitative data that followed normal distribution were statistically described by mean ± standard deviation, and independent sample *t*-test was used for the analysis of intergroup variation. Data that did not follow a normal distribution were statistically described by median (interquartile range) and intergroup variability was assessed using a nonparametric test. Qualitative data were statistically described by percentages, and χ^2^ test was used for the analysis of intergroup variation. In the single-factor analysis, the Variance Inflation Factor (VIF) and Tolerance (TOL) were used to determine whether there was exact linear dependence among the variables. The variables that were statistically significant in the single-factor analysis and the related variables reported in the literature were analyzed using a backward stepwise regression with quasi-maximum likelihood estimation (*α*_*1 =*_ 0.05, α2 = 0.10) to establish a logistic regression analysis model, which was subsequently visualized using a nomogram (alignment diagram). The area under ROC was used to evaluate the distribution of the model and the Hosmer-Lemeshow *χ*^*2*^ test and calibration plots were used to evaluate the calibration of the model. The test level was α = 0.05.

## Results

### Baseline characteristics

A total of 669 patients undergoing colonoscopy [318 (47.5%) women and 351 (52.5%) men] of ages 50.80 ± 13.78 years were included in the modeling group. In this group, 162 (24.2%) patients failed bowel preparation. A total of 446 patients undergoing colonoscopy [236 (52.9%) women and 210 (47.1%) men] of 50.50 ± 13.73 years were included in the validation group; 108 (24.2%) patients in this group failed bowel preparation.

### Single-factor analysis of bowel preparation failure in the modeling group

Single-factor analysis showed statistically significant differences (*P* < 0.05) between patients of different ages, sex and body mass index (BMI ≥ 24 kg/m^2^), defecation frequency, smoking, alcohol consumption, chronic constipation, diabetes mellitus, and history of colorectal surgery. (Table 1)

**Table 1:** Single-factor analysis of failed bowel preparation in patients undergoing colonoscopy

	Bowel preparation success group (*n* = 507)	Bowel preparation failure group (*n* = 162)	χ^2^	*P*
Age (years, y)			4.776	0.029
<60	368(72.6)	103(63.6)		
≥ 60	139(27.4)	59(36.4)		
Sex			7.353	0.007
Female	256(50.5)	62(38.3)		
Male	251(49.5)	100(61.7)		
BMI ≥ 24 kg/m^2^			7.260	0.007
No	296(58.4)	75(46.3)		
Yes	211(41.6)	87(53.7)		
Examination time			1.922	0.166
Morning	495(58.6)	171(63.3)		
Afternoon	350(41.4)	99(36.7)		
Education level			4.654	0.325
Elementary school and below	37(7.3)	15(9.3)		
Junior High School	136(26.8)	37(22.8)		
High School	96(18.9)	36(22.2)		
College	109(21.5)	26(16.0)		
Bachelor’s degree and above	129(25.4)	48(29.6)		
Dietary preferences			0.591	0.744
Meat and vegetables	409(80.7)	135(83.3)		
Vegetarian-based	81(16.0)	22(13.6)		
Meat-based	17(3.4)	5(3.1)		
Exercise habits			0.296	0.863
Never exercise	51(10.1)	14(8.6)		
Occasional exercise	246(48.5)	79(48.8)		
Regular exercise	210(41.4)	69(42.6)		
Smoking			8.753	0.003
No	399(78.7)	109(67.3)		
Yes	108(21.3)	53(32.7)		
Drinking			5.671	0.017
No	397(78.3)	112(69.1)		
Yes	110(21.7)	50(30.9)		
Defecation frequency			9.242	0.010
<3	66(13.0)	37(22.8)		
3 ~ 14	331(65.3)	96(59.3)		
>14	110(21.7)	29(17.9)		
Indication			5.327	0.377
Abdominal discomfort	148(29.2)	45(27.8)		
Change in defecation frequency	90(17.8)	28(17.3)		
Occult blood in stool or blood in the stool	52(10.3)	9(5.6)		
Follow-up	103(20.3)	33(32.9)		
Medical examination	80(15.8)	32(27.1)		
Other	34(6.7)	15(9.3)		
Comorbidities				
Chronic constipation	75(14.8)	40(24.7)	8.45	0.004
High blood pressure	88(17.4)	31(19.1)	0.266	0.606
Diabetes mellitus	23(4.5)	16(9.9)	6.377	0.012
Stroke	13 (2.6)	8(4.9)	2.276	0.131
Cirrhosis of the liver	11(2.2)	6(4.1)	0.629*	0.428
Use of opioids			0.405	0.524
No	495(97.6)	156(96.3)		
Yes	12(2.4)	6(3.7)		
Use of antidepressants			0.163	0.686
No	499(98.4)	158(97.5)		
Yes	8(1.6)	4(2.5)		
History of abdominal surgery			0.013	0.911
No	356(70.2)	113(69.8)		
Yes	113(29.8)	49(30.2)		
History of colorectal surgery			13.099	<0.001
No	477(94.1)	138(85.2)		
Yes	30(5.9)	24(14.8)		
Gastrectomy/Duodenectomy			0.906	0.341
No	485(95.7)	152(93.8)		
Yes	22(4.3)	10(6.2)		
Appendectomy			1.155	0.283
No	462(91.1)	143(88.3)		
Yes	45(8.9)	19(11.7)		
Hysterectomy			0.001	0.980
No	488(96.3)	156(96.3)		
Yes	19(3.7)	6(3.7)		
Bowel preparation failure history			0.376	0.540
No	493(97.2)	156(96.3)		
Yes	14(2.8)	6(3.7)		

BMI, body mass index

### Multifactorial analysis of bowel preparation failure in the modeling group

A covariance analysis was conducted on the statistically significant correlates of the single factor analysis and no exact linear dependence was found between variables with variance inflation factors of < 5 and tolerance values > 0.1 for each independent variable. Stepwise logistic regression analysis was conducted using variables with statistically significant differences in the single-factor analysis as well as related variables reported in the literature. The findings indicated that age ≥ 60 years, male sex, BMI ≥ 24 kg/m^2^, smoking, chronic constipation, diabetes mellitus, and history of colorectal surgery were independent risk factors for bowel preparation failure. (Table 2)

**Table 2:** Results of multifactorial analysis of failed bowel preparation in patients undergoing colonoscopy

Predictive variables	Beta value	Standard error	Wald χ^2^	*P*	OR value (95%CI)	
Constant term	-3.161	0.294	115.465	<0.001		—
Age ≥ 60 years	0.809	0.215	14.179	<0.001		2.246 (1.474, 3.423)
Male	0.895	0.261	11.816	0.001		2.449 (1.469, 4.080)
BMI ≥ 24 kg/m^2^	0.838	0.211	15.817	<0.001		2.311 (1.529, 3.492)
Smoking	0.903	0.248	13.299	<0.001		2.467 (1.519, 4.009)
Chronic constipation	1.649	0.291	32.172	<0.001		5.199 (2.941, 9.190)
Diabetes mellitus	1.686	0.399	17.852	<0.001		5.396 (2.469, 11.793)
History of colorectal surgery	1.643	0.329	24.949	<0.001		5.170 (2.713, 9.850)

BMI, body mass index

### Construction of a prediction model for the risk of bowel preparation failure

**Fig. 1:**
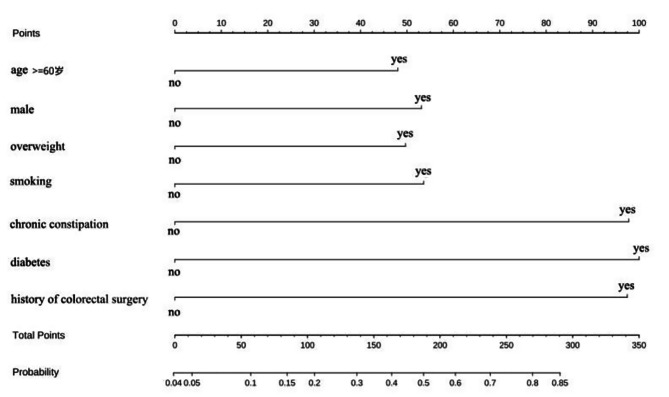
Alignment diagram prediction model for risk of bowel preparation failure in patients undergoing colonoscopy

An alignment diagram was developed for bowel preparation failure in patients undergoing colonoscopy based on the results of the multifactorial analysis. This was achieved by fitting a logistic model with selected variables as predictors (age ≥ 60 years, male sex, BMI ≥ 24 kg/m^2^, smoking, chronic constipation, diabetes mellitus, and history of colorectal surgery) and bowel preparation failure as the outcome event, as shown in Fig. 1. Using the alignment diagram, the scores for each predictor were summated to obtain the total score, and a straight line was drawn vertically downward from the total score to the last row to determine the predicted probability of bowel preparation failure occurring.

### Evaluation of the risk prediction model

**Fig. 2:**
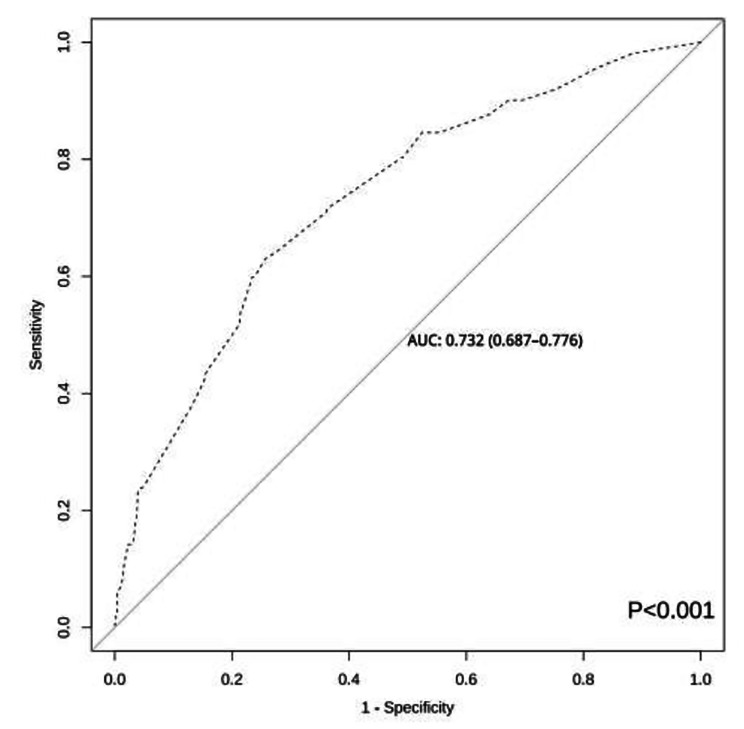
Receiver operating characteristic curves for the modeling group prediction model

**Fig. 3:**
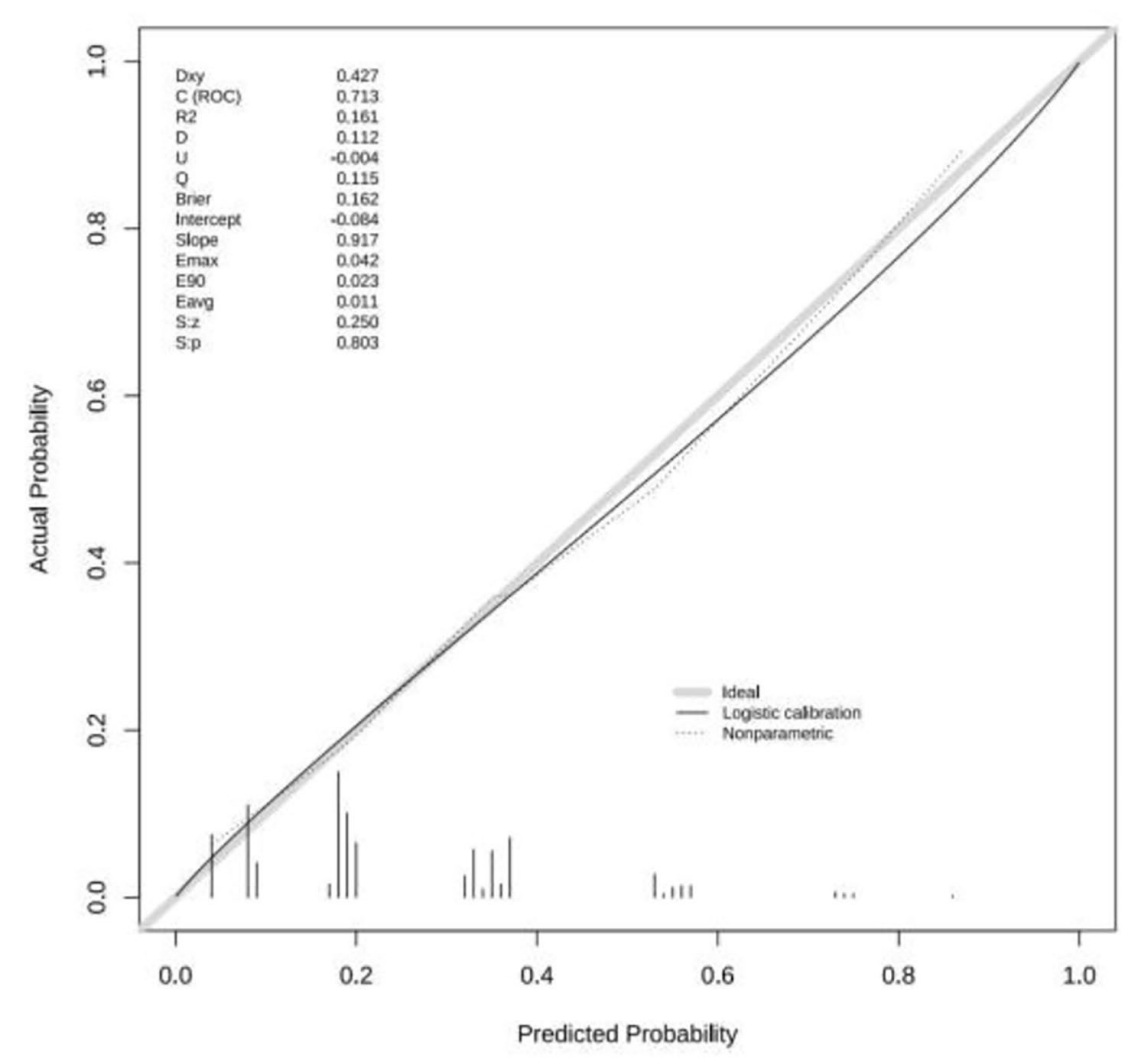
Calibration plot of the modeling group prediction model

**Fig. 4:**
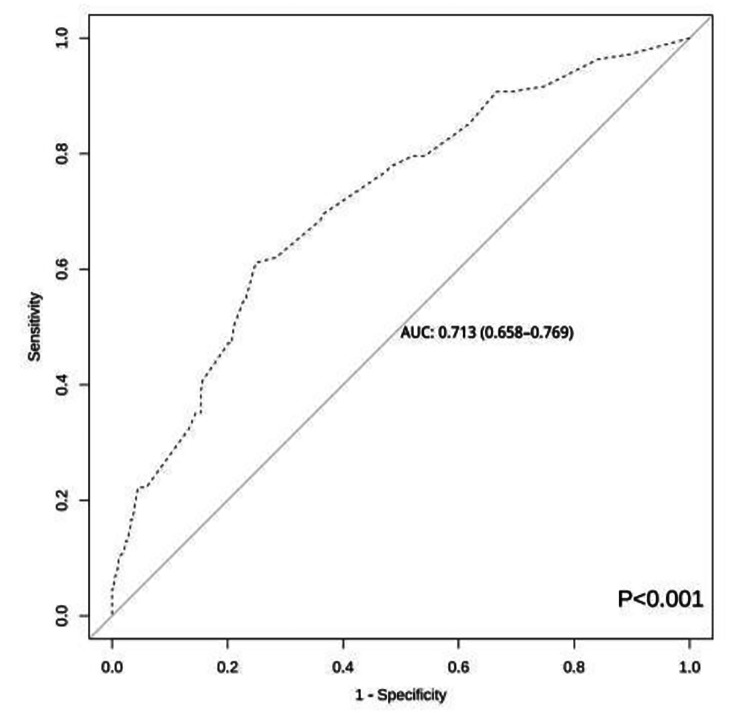
Receiver operating characteristic curves for the validation group prediction model

**Fig. 5:**
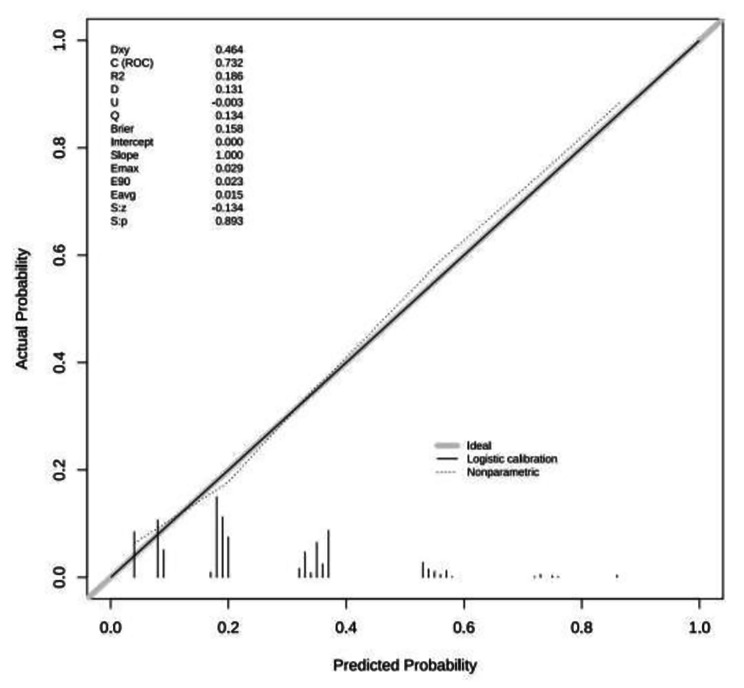
Calibration plot of the validation group prediction model

The area under the ROC curve of this prediction model was 0.732 (95% CI: 0.687 to 0.776, *P* < 0.001) and the maximum value of the Youden index was 0.373. The optimal cut-off value of 0.331 was considered the risk threshold and corresponded to a sensitivity of 62.96% and a specificity of 74.36% (Fig. 2). Hosmer-Lemeshow χ^2^ = 4.136, *p* = 0.845, and the calibration plot showed that the actual and ideal curves well overlapped (Fig. 3). The internal validation results showed that the area under the ROC curve of the model was 0.713 (95% *CI* 0.658–0.769, *p* < 0.001), the sensitivity of the model was 61.11%, the specificity was 75.15%, and the correct rate was 71.75% (Fig. 4). Hosmer-Lemeshow χ^2^ = 3.790 and *P* = 0.876. A good agreement was observed between the predictive effect of the model and the actual results in the calibration plot (Fig. 5).

## Discussion

Colonoscopy is the most effective method for screening for colon cancer and precancerous lesions. However, the effectiveness of colonoscopy is highly dependent on the quality of bowel preparation. To identify patients at high risk of bowel preparation failure, it is necessary to explore and analyze the risk factors for bowel preparation failure in colonoscopy patients and to develop an effective and distributive prediction model. Consequently, high-risk patients can receive intensive protocols and interventions, while low-risk patients can be prevented from taking excessive amounts of laxatives, thereby improving bowel preparation without increasing adverse effects.

In this study, patients aged ≥ 60 years were more likely to fail bowel preparation, and this has been associated with declining gastrointestinal motility, frequent concomitant disease and medication history, or limited or poorly tolerated physical activity. Comorbidities and medication history are the leading causes of bowel preparation failure among elderly patients, compared to other factors attributed to age [16]. In this study, the covariance analysis of age with related factors showed no significant covariance between age and other factors. Therefore, age ≥ 60 years was an independent risk factor for bowel preparation failure in patients undergoing colonoscopy.

Men had an independent risk factor for bowel preparation failure. Baker et al. [17] con ducted a retrospective study on 28,725 patients. Higher incidence of bowel preparation failure was observed in men than in women (56.6% vs. 43.4%), and men were associated with bowel preparation failure (*OR* = 1.353). This could be attributed to poorer compliance with bowel preparation protocols by men. In this study, women were more attentive to bowel preparation details during colonoscopy appointments than men and repeatedly confirmed with the appointment staff the dietary choices and timing of laxative administration in the bowel preparation protocol. Therefore, health education should be enhanced for male patients scheduling colonoscopies to emphasize the importance of bowel preparation prior to the examination. This can ensure patients restrict their diet and adhere to laxatives administration timing.

BMI ≥ 24 kg/m^2^ was an independent risk factor for bowel preparation failure in patients undergoing colonoscopy. Similarly to the results of this study, Borg et al. [18] showed that patients with a BMI > 25 kg/m^2^ had a greater risk of bowel preparation failure. A prospective study by Sharara et al. [19] showed that a BMI < 20 kg/m^2^ (underweight) was a risk factor for bowel preparation failure. In a prospective study conducted by Fok et al. [20], no statistically significant difference was found between obese and non-obese patients regarding bowel preparation. Oral sodium picosulfate was used in their study. The differences between the results of the above studies and the current study may be attributed to the different study populations, study designs, BMI classification criteria, and bowel preparation procedures. In this study, according to the BMI classification standard of Chinese population, BMI ≥ 24 kg/m^2^ was considered overweight. The findings of this study showed that overweight patients are more likely to experience bowel preparation failure, possibly because of physical inactivity, chronic constipation, and multiple chronic illnesses.

It was also observed that smokers are more likely to fail bowel preparation. When smoking results in greater sympathetic excitability, bowel movements are slowed. Moreover, chronic smoking is associated with a number of diseases and poses high risk of comorbidity. Furthermore, smokers are less likely to be health conscious and less likely to comply with bowel preparation protocols. Accordingly, patients who smoke should be encouraged to quit smoking and health education should be provided during bowel preparation.

Constipation was found to be an independent risk factor for the failure of bowel preparation before colonoscopy. This is consistent with the findings of Gimeno-García et al. [11] In a prospective cohort study of 409 patients undergoing colonoscopy, Fang et al. [21] also identified chronic constipation as an independent risk factor for bowel preparation failure (*OR* = 2.05). As a result of decreased autonomic nervous system function, relaxation, slowed peristalsis, and reduced activity of the intestinal muscles, constipated patients are more likely to experience prolonged bowel emptying times and weakness in defecation. This increases the amount of feces remaining in the intestinal lumen and adversely affects the quality of bowel preparation. In China, chronic constipation affects 4–10% of adults and shows an upward trend. Therefore, healthcare professionals are advised to follow up with patients experiencing constipation after undergoing colonoscopy. It is recommended that patients with constipation eat a low residue diet 3 days prior to the examination and take high-dose bowel cleansing medications and adjunctive bowel medications as prescribed by their physician in order to reduce the likelihood of bowel preparation failure.

Patients with diabetes mellitus are more likely to experience failure of bowel preparation. As gastric motility and emptying are regulated by blood glucose, an increase in blood glucose inhibits gastric emptying and slows down gastrointestinal transport, and diabetes mellitus in later stages leads to peripheral and autonomic neuropathy, resulting in abnormal gastrointestinal function in diabetics, which can lead to constipation in 90% of cases. While Izzy et al. [22] reported that bowel preparation failure in diabetic patients was not associated with short-term glycemic control. Further research is needed to examine the relationship between glycemic control and bowel preparation in diabetics.

In terms of the relationship between previous abdominal surgical history and the quality of bowel preparation, no definitive conclusion was made in this study. According to Chung et al. [23], detailed classification of prior surgical history, appendectomy, hysterectomy, and colorectal resection were independent risk factors for bowel preparation failure. While a history of abdominal surgery was not found to be an important factor in bowel preparation failure in Cheng et al. [16], possibly because the study did not clearly define the various types of abdominal surgery. This study examined the relationship between abdominal surgery, colorectal surgery, gastric/duodenectomy, appendectomy, and hysterectomy, and the quality of bowel preparation in colonoscopy patients. A history of colorectal surgery was found to be a risk factor independently associated with failure of bowel preparation. Owing to altered intestinal anatomy and adhesions, patients after colorectal surgery experienced reduced bowel motility and emptying ability, and intestinal lumen contents remained in their bowels, thereby reducing the quality of their bowel preparation [24]. Therefore, healthcare professionals can encourage patients undergoing colonoscopy with a history of colorectal surgery to engage in moderate-intensity aerobic exercise, such as walking, and massage their abdomen during medication administration to promote intestinal peristalsis, in addition to a comprehensive bowel preparation program.

The prediction model for the risk of failure of bowel preparation established in this study showed a good predictive effect. The areas under the ROC curves of the modeling and validation groups were 0.732 and 0.713, respectively. The Hosmer-Lemeshow χ^2^ test for the prediction model was *P* > 0.05. The calibration plots showed good agreement between the model prediction probability and the actual occurrence probability. The areas under the ROC curve ranged from 0.63 to 0.72 in the published studies [9–11]. The model of this study has been presented as an alignment diagram, which is easier to visualize and is convenient for clinical use. When patients schedule appointments for colon examinations, the medical staff at the endoscopy center can use this alignment diagram to predict the probability of failure of the bowel preparation process and provide personalized bowel preparation plans. Notwithstanding its success, this study has limitations. This was a single-center study and the model used had only internal validation. A multicenter and large-sample studies will be necessary to validate the model externally and assess its predictive ability.

## Conclusions

Age ≥ 60 years, male sex, BMI ≥ 24 kg/m^2^, smoking, chronic constipation, diabetes mellitus, and history of colorectal surgery are independent risk factors for bowel preparation failure in patients undergoing colonoscopy. The alignment diagram for predicting the risk of bowel preparation failure constructed in this study has excellent predictive efficacy and can be clinically applied to identify patients at high risk of bowel preparation failure when making appointments for colonoscopy.

## Data Availability

The data presented in this study are openly available in Science Data Bank at https://www.scidb.cn/en/s/amyeym.

## Abbreviations

ROC: Receiver operating characteristic
BMI: Body mass index
BBPS: Boston Bowel Preparation Scale
